# A Retrospective Comparative Study of the Outcomes of Single-Stage Versus Double-Stage Bilateral Total Knee Arthroplasty in the Management of Bilateral Knee Osteoarthritis

**DOI:** 10.7759/cureus.84266

**Published:** 2025-05-17

**Authors:** Borusu Siva Narayana, Nagakumar J S, Sagar Venkataraman, Gils Thampi

**Affiliations:** 1 Orthopedics, Sri Devaraj Urs Medical College, Sri Devaraj Urs Academy of Higher Education and Research, Kolar, IND

**Keywords:** double-stage tka, functional outcomes, knee replacement, osteoarthritis, perioperative complications, single-stage tka, total knee arthroplasty

## Abstract

Background

Osteoarthritis of the knee is a prevalent chronic condition, often necessitating total knee arthroplasty (TKA) when conservative treatments fail. Single-stage and double-stage bilateral TKA procedures are both widely performed, and the optimal approach in terms of perioperative complications and functional outcomes remains debated. This study aims to compare single-stage and double-stage bilateral TKA concerning postoperative recovery, complications, and hospitalization parameters.

Methods

A retrospective study was conducted at R. L. Jalappa Hospital, Tamaka, Kolar, from August 2023 to July 2024, including 38 patients with Kellgren-Lawrence grade 3 or 4 primary osteoarthritis undergoing bilateral TKA. Patients were divided into two cohorts: Group A (n = 19) underwent single-stage TKA, and Group B (n = 19) underwent double-stage TKA (≤12 months apart). Outcomes assessed included pain (Visual Analogue Scale (VAS)), functional recovery (Oxford Knee Score (OKS), Knee Society Score (KSS)), perioperative complications, transfusion rates, and hospitalization duration. Statistical analyses compared both groups.

Results

The mean age of participants was comparable (65.74 ± 8.13 years in Group A vs. 65.58 ± 10.01 years in Group B). Both groups exhibited similar preoperative VAS (8.53 ± 1.17 vs. 8.47 ± 0.96), OKS (20.32 ± 3.20 vs. 19.74 ± 3.21), and KSS (50.58 ± 5.09 vs. 50.11 ± 5.5). The single-stage group had a significantly shorter hospital stay (6.21 ± 0.85 days vs. 7.74 ± 1.28 days, p = 0.001). Functional scores at six months showed higher OKS in Group A (50.00 ± 3.23) than Group B (47.95 ± 2.697, p = 0.040), while KSS scores were similar. Perioperative complications included deep vein thrombosis (DVT) in three (7.8%) patients, bleeding in one (2.6%) patient, pulmonary embolism (PE) in one (2.6%) patient, wound healing issues in two (5.3%) patients, and urinary complications in two (5.3%) patients, with no significant difference between groups.

Conclusion

Single-stage bilateral TKA is associated with shortened hospital stay and improved functional outcomes at six months compared to the double-stage approach, without significant differences in perioperative complications. These findings suggest that single-stage TKA may be a preferable option in carefully selected patients.

## Introduction

Osteoarthritis most commonly affects the knee, which affects nearly four out of five individuals globally [1]. Osteoarthritis is an irreversible, persistent condition that gradually outgrows conservative therapies, including medication and physical therapy. Surgery to replace the joint is still the only way to manage symptoms and enhance a patient's quality of living. These days, total knee arthroplasty (TKA) is a frequent orthopedic procedure used to treat severe osteoarthritis or systemic diseases, a technique to restore function [2].

Radiography is mostly used for diagnosis. One of the most popular radiographic classifications for determining the stage of osteoarthritis in the knee is the Kellgren-Lawrence classification, which uses morpho-structural alterations to categorize osteoarthritic joints into four severity levels [3]. Over the past few decades, TKA indications have been steadily increasing. Among the primary causes of the unstoppable rise in these implants are longer life expectancies, significant variations in functional requirements, particularly among older cases, and improvements in surgical and anesthesiology methods. Bilateral osteoarthritis has a 5% incidence rate. A prosthetic knee on the opposite side procedure is necessary in the years that follow for one-third of individuals who have knee prosthesis surgery [4]. Patients who need bilateral knee replacements have two options: a "one-stage" procedure, which involves replacing both knees with a single hospital stay and surgical procedure, and a "two-stage" procedure, which involves the patient being hospitalised twice and undergoing two different surgical techniques over time.

There will be disagreement in the available literature regarding which of the two treatments is better for the patient's recuperation and safer. Because of a reported increase in perioperative complications, such as wound complications, pulmonary embolism (PE), deep vein thrombosis (DVT), and cardiac and neurological issues, which have led to a higher need for admissions to the intensive care unit, perioperative morbidity and mortality rates in simultaneous bilateral knee replacement surgery remain controversial. However, other research has shown that multistage treatments have a higher rate of problems [5-7]. Our study's objective is to determine whether, in terms of functional outcomes, a one-stage surgery is superior to a technique involving two distinct procedures. Perioperative complications were also assessed.

## Materials and methods

Study design

This retrospective study was conducted at R. L. Jalappa Hospital, Tamaka, Kolar, from August 2023 to July 2024.

Study population and sample size

A total of 38 patients undergoing bilateral total knee replacement are evaluated. Two cohorts were compared: Group A (n = 19) underwent single-stage surgery, while Group B (n = 19) underwent double-stage surgery (≤12 months between procedures). Surgeries were performed by the same surgeon, and data were collected from the hospital’s computerized system. Patients aged 40-75 years with Kellgren-Lawrence grade 3 or 4 primary osteoarthritis were included, while those with unicompartmental knee replacements, complicated prostheses, secondary arthritis, or coagulation disorders were excluded. The sample size was calculated using prevalence data (5%), yielding 19 patients per group.

Study measures

Our study aimed to determine if a single-stage surgery can be regarded as noninferior to a method involving two distinct surgeries in terms of functional results. Additionally, we assessed perioperative complications. Follow-ups were conducted at one, three, and six months, assessing pain using the visual analogue scale (VAS), function using the Oxford Knee Score (OKS) and Knee Society Score (KSS), and complications such as infections, transfusions, and adverse events based on the Patient Blood Management (PBM) guidelines. 

Ethics statement

Ethical clearance was received from the institutional ethical committee of Sri Devraj Urs Medical College (approval number SDUAHER/KLR/R&D/CEC/S/PG/134/2024-25) to conduct the research. 

Statistical analysis 

Data was collected in a semi-structured questionnaire and entered into MS Excel (Microsoft Corporation, Redmond, Washington, United States. Data was analyzed by using IBM SPSS Statistics for Windows, Version 26 (Released 2019; IBM Corp., Armonk, New York, United States). Qualitative data were described as frequencies and percentages, and quantitative data were described as mean and SD. To analyze various variables, the chi-square test, the t-test, and the analysis of variance (ANOVA) test will be used. A p-value of less than 0.05 is considered statistically significant.

## Results

The mean age of participants in Group A (single-stage procedure) was 65.74 years (SD = 8.13), while the mean age in Group B (double-stage procedure) was 65.58 years (SD = 10.01). The BMI of participants was comparable between the groups, with Group A reporting a mean BMI of 28.38 kg/m² (SD = 3.38) and Group B reporting a mean BMI of 28.39 kg/m² (SD = 3.85) (Table 1).

**Table 1:** Characteristics of study participants (N = 38) BMI: body mass index Note: Values are reported as mean ± standard deviation

Variables	Single-stage (Group A) (n = 19)	Double-stage (Group B) (n = 19)
Age (years)	65.74 ± 8.13	65.58 ± 10.01
BMI (kg/m²)	28.38 ± 3.38	28.39 ± 3.85

This study included 38 patients undergoing bilateral total knee replacement, with 19 patients in each group (single-stage vs. double-stage procedures). The gender distribution was nearly balanced, with 20 females and 18 males. The need for transfusion was observed in six patients (15.8%), while 32 patients (84.2%) did not require transfusion. Postoperative complications were reported in nine patients (23.7%), with DVT in three patients (7.9%), bleeding in one patient (2.6%), PE in one patient (2.6%), wound healing issues in two patients (5.3%), and urinary complications in two patients (5.3%). Regarding comorbidities, diabetes was the most prevalent (10 patients, 26.3%), followed by hypertension (eight patients, 21.1%), cardiac disease (four patients, 10.5%), chronic kidney disease (CKD) (three patients, 7.9%), and obesity (two patients, 5.3%) (Table 2).

**Table 2:** Distribution of complications and comorbidities of study participants ( n = 38) DVT: deep vein thrombosis; PE: pulmonary embolism; CKD: chronic kidney disease

Variable	Category	N (%)
Group	A (single-stage)	19 (50.0)
	B (double-stage)	19 (50.0)
Gender	Female	20 (52.6)
	Male	18 (47.3)
Transfusion Required	No	32 (84.2)
	Yes	6 (15.7)
Complications	None	29 (76.3)
	DVT	3 (7.8)
	Bleeding	1 (2.6)
	PE	1 (2.6)
	Wound healing	2 (5.2)
	Urinary issues	2 (5.2)
Comorbidities	Diabetes	10 (26.3)
	Hypertension	8 (21.0)
	Cardiac disease	4 (10.5)
	CKD	3 (7.8)
	Obesity	2 (5.2)

The current study analyzed preoperative clinical scores of 38 patients, with 19 in the single-stage group (Group A) and 19 in the double-stage group (Group B). Preoperatively, pain intensity, as assessed using the VAS, was comparable between the two groups (8.53 ± 1.17 in Group A vs. 8.47 ± 0.96 in Group B). Functional assessment using the OKS indicated similar levels of knee function impairment (20.32 ± 3.20 in Group A vs. 19.74 ± 3.21 in Group B). The KSS, a measure of knee function and alignment, also showed minimal differences (50.58 ± 5.09 in Group A vs. 50.11 ± 5.5 in Group B). Regarding preoperative hemoglobin (g/dL) levels, Group A had a slightly higher mean value (13.04 ± 1.10 g/dL) compared to Group B (12.58 ± 0.99 g/dL). However, this difference was small and may not be clinically significant (Table 3).

**Table 3:** Preoperative clinical scores of study participants (n = 38) VAS: visual analogue scale; OKS: Oxford Knee Score; KSS: Knee Society Score Note: Values are reported as mean ± standard deviation. VAS (points), OKS (points), KSS (points), hemoglobin (g/dL)

Variables	Group A (mean ± SD)	Group B (mean ± SD)
VAS (pain, points)	8.53 ± 1.17	8.47 ± 0.96
Oxford Knee Score (points)	20.32 ± 3.20	19.74 ± 3.21
Knee Society Score (KSS, points)	50.58 ± 5.09	50.11 ± 5.50
Preop hemoglobin (g/dL)	13.04 ± 1.10	12.58 ± 0.99

The current study evaluated postoperative outcomes and hospitalization parameters in 38 patients, with 19 in the single-stage group (Group A) and 19 in the double-stage group (Group B). Group A had a significantly shorter mean hospital stay (6.21 ± 0.85 days) compared to Group B (7.74 ± 1.28 days), indicating that single-stage surgery was associated with reduced hospitalization duration.

Postoperative pain assessment revealed a steady decline in VAS scores over time in both groups. At one month, pain levels were similar (3.95 ± 0.84 in Group A vs. 3.95 ± 0.848 in Group B). By three months, pain scores slightly favored Group B (3.00 ± 0.81 in Group A vs. 2.63 ± 0.831 in Group B), and this trend continued at six months (2.26 ± 0.73 in Group A vs. 1.95 ± 0.780 in Group B).

The OKS improved in both groups, with comparable scores at one month (29.68 ± 3.14 in Group A vs. 29.53 ± 2.435 in Group B) and three months (39.95 ± 3.39 vs. 39.74 ± 2.705, respectively). At six months, Group A exhibited a slightly higher improvement (50.00 ± 3.23 vs. 47.95 ± 2.697 in Group B).

Similarly, the KSS showed progressive improvement over time. Both groups had comparable scores at one month (59.95 ± 5.69 in Group A vs. 58.89 ± 5.45 in Group B) and at three months (76.53 ± 4.59 vs. 77.32 ± 4.28, respectively). At six months, Group A had a marginally higher score (90.68 ± 2.88 vs. 89.63 ± 2.96 in Group B), indicating similar functional recovery. Postoperatively, hemoglobin (g/dL) levels decreased in both groups at one month (11.26 ± 1.144 in Group A vs. 10.842 ± 0.88 in Group B) but gradually improved over time. At three months, Group A had slightly higher levels (11.70 ± 1.103 vs. 11.347 ± 0.87 in Group B), and this pattern persisted at six months (12.02 ± 1.088 vs. 11.695 ± 0.89 in Group B) (Table 4).

**Table 4:** Hospitalization and postoperative outcomes (N = 38) VAS: visual analogue scale; OKS: Oxford Knee Score; KSS: Knee Society Score Note: Values are reported as mean ± standard deviation. Units: hospital stay (days), VAS (points), OKS (points), KSS (points), hemoglobin (g/dL)

Variables	Group A (mean ± SD)	Group B (mean ± SD)
Hospital stay (days)	6.21 ± 0.85	7.74 ± 1.28
VAS (pain, points): 1 month	3.95 ± 0.84	3.95 ± 0.848
VAS (pain, points): 3 months	m3.00 ± 0.81	2.63 ± 0.831
VAS (pain, points): 6 months	2.26 ± 0.73	1.95 ± 0.780
OKS (points): 1 month	29.68 ± 3.14	29.53 ± 2.435
OKS (points): 3 months	39.95 ± 3.39	39.74 ± 2.705
OKS (points): 6 months	50.00 ± 3.23	47.95 ± 2.697
KSS (points): 1 month	59.95 ± 5.69	58.89 ± 5.45
KSS (points): 3 months	76.53 ± 4.59	77.32 ± 4.28
KSS (points): 6 months	90.68 ± 2.88	89.63 ± 2.96
Hemoglobin (g/dL): 1 month	11.26 ± 1.144	10.842 ± 0.88
Hemoglobin (g/dL): 3 months	11.70 ± 1.103	11.347 ± 0.87
Hemoglobin (g/dL): 6 months	12.02 ± 1.088	11.695 ± 0.89

A comparative analysis between single-stage and double-stage TKA showed no significant differences in age (p = 0.958), BMI (p = 0.989), or preoperative clinical scores (VAS, OKS, KSS, and haemoglobin; p > 0.05 for all).

Hospital stay was significantly shorter in the single-stage group (6.2 ± 1.3 days vs. 7.7 ± 1.3 days, p = 0.001). At 6 months, pain scores (VAS) were comparable between groups (p = 0.207), but functional outcomes (OKS) were significantly better in the single-stage group (p = 0.040). KSS and haemoglobin (g/dL) levels showed no statistically significant differences at 6 months (p = 0.275 and p = 0.319, respectively) (Table 5).

**Table 5:** Comparative analysis (single vs. double-stage TKA) TKA:  total knee arthroplasty; BMI: body mass index; OKS: Oxford Knee Score; KSS: Knee Society Score p < 0.01 indicates high statistical significance. Note: Values are reported as mean ± standard deviation. Units: age (years), BMI (kg/m²), VAS (points), OKS (points), KSS (points), hemoglobin (g/dL), and hospital stay (days). Where applicable, chi-square or t-tests were performed with degrees of freedom (df = 36). Effect sizes (Cohen's d) are included for significant results

Variable	Group A (Mean ± SD)	Group B (Mean ± SD)	p-value	Test Statistic (df) / Effect Size
Age (years)	65.7 ± 8.9	65.6 ± 9.1	0.958	t(36) = 0.05
BMI (kg/m²)	28.4 ± 3.5	28.4 ± 3.7	0.989	t(36) = 0.01
Pre-op VAS (points)	8.53 ± 1.1	8.47 ± 1.0	0.881	t(36) = 0.15
Pre-op OKS (points)	20.3 ± 3.2	19.7 ± 3.2	0.581	t(36) = 0.39
Pre-op KSS (points)	50.6 ± 5.3	50.1 ± 5.3	0.785	t(36) = 0.27
Pre-op Hemoglobin (g/dL)	13.0 ± 1.1	12.6 ± 1.1	0.183	t(36) = 1.36
Hospital Stay (days)	6.2 ± 1.3	7.7 ± 1.3	0.001*	t(36) = 4.26, d = 1.37
6M VAS (points)	2.26 ± 0.8	1.95 ± 0.8	0.207	t(36) = 1.27
6M OKS (points)	50.0 ± 3.1	47.9 ± 3.1	0.040*	t(36) = 2.15, d = 0.70
6M KSS (points)	90.7 ± 2.9	89.6 ± 2.9	0.275	t(36) = 1.11
6M Hemoglobin (g/dL)	12.0 ± 1.0	11.7 ± 1.0	0.319	t(36) = 1.01

## Discussion

The findings of this retrospective comparative study indicate that single-stage bilateral TKA offers several advantages over the two-stage approach, particularly in terms of reduced hospital stay and improved functional outcomes at the six-month follow-up. Having a p-value of 0.001 means that high statistical significance, patients in the single-stage group had a considerably shorter mean hospital stay (6.21 ± 0.85 in days) when compared to those in the double-stage unit (7.74 ± 1.28 days). This reduction in hospitalization time aligns with existing literature, where simultaneous bilateral TKA has been associated with decreased overall hospital stays and healthcare costs [8-11]. 

At the six-month mark, the single-stage group demonstrated superior functional outcomes, as evidenced by higher Oxford Knee Scores (OKS) (50.00 ± 3.23) compared to the double-stage group (47.95 ± 2.697), with a p-value of 0.040. This improvement is consistent with previous studies that have reported enhanced functional recovery among cases undergoing bilateral TKA that is synchronous [12-14]. 

The total prevalence of complications was comparable between the two groups. Six patients in the single-stage cohort needed revision because of problems such as patella lateralization, dislocation, fracture, peroneal nerve palsy, or loosening. On the first postoperative day, however, a quadriceps tendon rupture brought on by a fall only necessitated revision for one patient in the double-stage group [15-17]. 

In a study done by Mukherjee et al., a single intraoperative dose of tranexamic acid (TXA) may not be adequate to reduce blood loss and transfusion needs in single-stage bilateral TKA. TXA is commonly used to minimize blood loss and transfusion requirements. However, the optimal regimen of TXA in single-stage bilateral TKA is still not defined. In this retrospective study, 35 patients who received TXA and 31 patients who did not receive TXA were evaluated for blood loss and transfusion requirement. Both groups were comparable in terms of age, sex, BMI, and preoperative hemoglobin and hematocrit. There was no significant difference in the change in hemoglobin levels (2.42 ± 1.28 vs 2.44 ± 1.31; p = 0.95) and hematocrit (1.37 ± 0.96 vs 1.62 ± 0.98, p = 0.22) between the two groups [18-19]. 

This observation aligns with existing literature, which reports similar complication rates between single-stage and “two-stage bilateral TKA” procedures [9,19]. The study noted more patients in the single-stage group who needed blood transfusions, although precise information on blood loss and transfusion rates was not provided. This observation is consistent with previous research indicating that increased perioperative blood loss and transfusion needs may be linked to concomitant bilateral TKA [10,20]. 

Limitations

There are several restrictions on this study. Because of the small sample size, the results might not be as widely relevant as they could be. Furthermore, just one center was used for the study, which could have introduced selection bias. To evaluate long-term problems and functional outcomes, longer follow-up is required. The outcomes may also be impacted by potential confounders like comorbidities and preoperative fitness levels. To confirm these results, larger sample sizes are required in future multicenter randomized controlled study.

## Conclusions

This research found that single-stage bilateral TKA produced a noticeably shorter hospital stay and better functional outcomes at six months compared to the two-stage approach. Both groups experienced significant pain reduction, with no statistically significant difference in postoperative pain scores. While complication rates were comparable, the single-stage group had a higher requirement for blood transfusions. Overall, single-stage bilateral TKA appears to be a safe and efficient option for appropriately selected patients, though careful preoperative planning is necessary.
